# 6PPD-Quinone Induces Anxiety-like Behavior in Mice: A Potential Mechanism Shared Across PPD-Quinones

**DOI:** 10.3390/toxics14100909

**Published:** 2026-10-14

**Authors:** Su Xu, Leyan Gao, Yongquan Yu, Ying Kang, Xinting Chen, Shou-Lin Wang, Zengliang Ruan

**Affiliations:** 1 Key Laboratory of Environmental Medicine and Engineering of Ministry of Education, School of Public Health, Southeast University, Nanjing 210009, China; 220234045@seu.edu.cn (S.X.); 101013071@seu.edu.cn (Y.Y.); 220254161@seu.edu.cn (Y.K.); 220244211@seu.edu.cn (X.C.); 2 State Key Lab of Reproductive Medicine and Offspring Health, Institute of Toxicology, School of Public Health, Nanjing Medical University, Nanjing 211166, China; gaoleyan@stu.njmu.edu.cn

**Keywords:** 6PPD-Q, PPD-Qs, anxiety-like behavior, cAMP signaling, ADORA2A

## Abstract

The increasing occurrence of anxiety disorders caused by emerging environmental toxicants has become a significant global health concern. While recent evidence indicates that 6PPD-Q can induce anxiety-like behavior, the specific mechanisms connecting 6PPD-Q to anxiety-like behavior are still largely unknown. This gap in understanding restricts our knowledge of their neurotoxic potential and complicates the development of targeted treatment strategies. Here, adverse effects, including neuronal degeneration, synaptic dysfunction, and impaired GABAergic signaling in the amygdala, were observed in mice exhibiting anxiety-like behavior following 6PPD-Q exposure. Integrated network toxicology and transcriptomic analysis revealed that inhibition of the cAMP signaling pathway was associated with PPD-Q-related anxiety-like behavior, and adenosine receptors, particularly ADORA2A, as potential targets of 6PPD-Q. Specifically, molecular docking and molecular dynamics simulations suggested that 6PPD-Q may stably interact with the orthosteric ligand-binding pocket of ADORA2A, potentially interfering with receptor activation. Importantly, the predicted interaction with ADORA2A may represent a potentially shared neurotoxicological feature among PPD-Qs, consistent with the computationally predicted interaction profiles across PPD-Qs. Moreover, pharmacological activation of ADORA2A with the selective agonist significantly alleviated the 6PPD-Q-induced neurobehavioral deficits in mice. Collectively, our research provides evidence supporting the involvement of ADORA2A and the cAMP-related signaling pathway in 6PPD-Q-induced anxiety-like behavior, providing a timely foundation for preventing neuropsychiatric disorders associated with PPD-Q exposure.

## 1. Introduction

The rising global burden of neuropsychiatric disorders has become a critical public health challenge. It is estimated that mental disorders affect approximately 22% of the global population, with anxiety-like behavior accounting for a substantial proportion of this burden and ranking as the sixth leading cause of disability in both high- and low-income countries [1,2,3]. These conditions profoundly impair daily functioning and erode overall quality of life, limiting individuals’ capacity to sustain work productivity, maintain social relationships, and engage in routine activities. This functional deterioration, in turn, generates substantial economic repercussions, fueled by elevated healthcare utilization and productivity losses attributable to both absenteeism and presenteeism [4,5]. Indeed, diminished workforce output stemming from mental disorders currently drains the global economy of US$1 trillion annually, a sum expected to rise to US$16 trillion by 2030 [6]. This burden is further compounded by the frequent co-occurrence of anxiety with a range of chronic non-communicable conditions, as a common and burdensome comorbidity rather than merely a predisposing factor [7]. Therefore, given the substantial prevalence and economic and health tolls of anxiety-like behavior, advancing our understanding of their causes is imperative. This knowledge is vital for identifying modifiable risk factors, which forms the scientific cornerstone for developing effective, targeted prevention strategies to alleviate the global burden. However, the etiology of anxiety-like behavior is complicated, involving not only genetics, family history, socioeconomic status, and medical conditions, but also a significant contribution from environmental factors [8,9]. Indeed, mounting evidence from recent studies across diverse global regions has repeatedly linked exposure to emerging environmental pollutants with an increased risk of anxiety [10,11,12], underscoring environmental exposure as a critical and modifiable component of the disease’s etiology.

The rapid expansion of the automotive industry has driven a massive increase in global tire production, making tire-derived chemicals a major source of environmental contamination [13]. p-phenylenediamine (PPD) antioxidants are extensively added to tires to retard aging. However, owing to their low molecular weight and aromatic amine structure, PPDs can be released into the environment from products during use, where they are then transformed into quinone derivatives (PPD-Qs) by ozone [14,15]. The environmental impact of these transformation products was long overlooked until 2021, when 6PPD-Q was established as the causative toxicant for acute urban runoff mortality in coho salmon, a discovery that sparked extensive research into the distribution and toxicology of this class of compounds [16]. Beyond acute ecotoxic effects, growing evidence has highlighted the role of 6PPD-Q in neural toxicity across multiple biological systems. It can induce oxidative stress, neuroinflammation, apoptosis, and mitochondrial dysfunction in the brain, disrupting neurotransmitter homeostasis and leading to behavioral abnormalities [17,18]. Specifically, chronic 6PPD-Q exposure has been shown to compromise the amygdalar function and induce anxiety-like behavior in mice, suggesting its potential to contribute to anxiety-related behavioral abnormalities [19]. However, the underlying mechanisms through which 6PPD-Q induces these adverse effects remain largely unknown.

A pivotal mechanism in environmental toxicant-induced anxiety-like behavior involves the suppression of cyclic adenosine monophosphate (cAMP), a master second messenger that triggers several downstream cellular mechanisms [20,21]. This disruption not only impairs the regulation of key factors such as brain-derived neurotrophic factor (BDNF) and neuropeptide Y but also extends to dysfunctions in multiple anxiety-related systems, including neural transmission, stress response, and immune-neural communication [22,23,24]. Mechanistically, intracellular cAMP homeostasis is tightly regulated through balanced synthesis and degradation. On one hand, synthesis is catalyzed by adenylyl cyclase (AC), which is directly activated or inhibited by Gs- or Gi-coupled GPCRs, respectively. On the other hand, its concentration is ultimately determined by degradation via phosphodiesterases (PDEs), which hydrolyze cAMP to inactive 5′-AMP [25,26]. Therefore, if 6PPD-Q decreases cAMP production and ultimately contributes to anxiety-like behavior in mice, this effect may be mediated by interactions between PPD-Qs and these GPCRs or PDEs. However, how 6PPD-Q modulates cAMP levels and downstream signaling pathways has yet to be investigated. This gap significantly limits the identification of specific biomarkers and therapeutic targets for mitigating the mental health risks posed by PPD-Q pollutants.

Herein, we hypothesize that 6PPD-Q may contribute to anxiety-like behavior through suppression of cAMP signaling and interactions with key regulatory targets. To test this hypothesis, we established an experimental model of 6PPD-Q-induced anxiety-like behavior in mice. We comprehensively investigated the accumulation of 6PPD-Q in the brain, its effects on anxiety-like behavior, and associated histopathological changes in the amygdala. The mechanisms underlying these effects were further evaluated using integrated transcriptomic, network toxicology and molecular docking analyses. These complementary approaches were used to investigate the potential molecular targets and signaling pathways associated with 6PPD-Q-induced anxiety-like behavior and to explore whether similar molecular patterns may occur among other PPD-Qs.

## 2. Materials and Methods

### 2.1. Reagents and Materials

6PPD-Q (97% purity, P348790) and its deuterium-labeled analog, 6PPD-Q-d5 (95% purity, P348791), were purchased from Toronto Research Chemicals (Toronto, ON, Canada). The selective adenosine A2A receptor (ADORA2A) agonist CGS 21680 hydrochloride (HY-13201A) and dimethyl sulfoxide (DMSO, HY-Y0320C) were purchased from MedChem Express (Monmouth Junction, NJ, USA). Olive oil (IO9000) was obtained from Solarbio (Beijing, China). Minimum Essential Medium (MEM) containing NEAA (10370088) and fetal bovine serum (FBS, 10091148) were purchased from Thermo Fisher Scientific (Waltham, MA, USA). Streptomycin and penicillin (C0222) and CCK-8 assay kit (C0038) were purchased from Beyotime Institute of Biotechnology (Shanghai, China). Commercial cAMP ELISA Kit (E-EL-0056) was obtained from Elabscience^®^ (Wuhan, China). The antibodies used in this study are listed in Table A1.

### 2.2. Animals and Treatment

Pathogen-free male C57BL/6 mice (8 weeks old; 23–27 g) were obtained from GemPharmatech Co., Ltd. (Shanghai, China) and maintained under specific pathogen-free (SPF) conditions at the Experimental Animal Center of Southeast University. They were allowed one week to acclimate to the environment before experiments began. The study was conducted in strict accordance with the institutional and national guidelines for animal welfare. All experimental protocols received prior approval from the Ethics Committee of Southeast University (20241011001).

**Figure 1:**
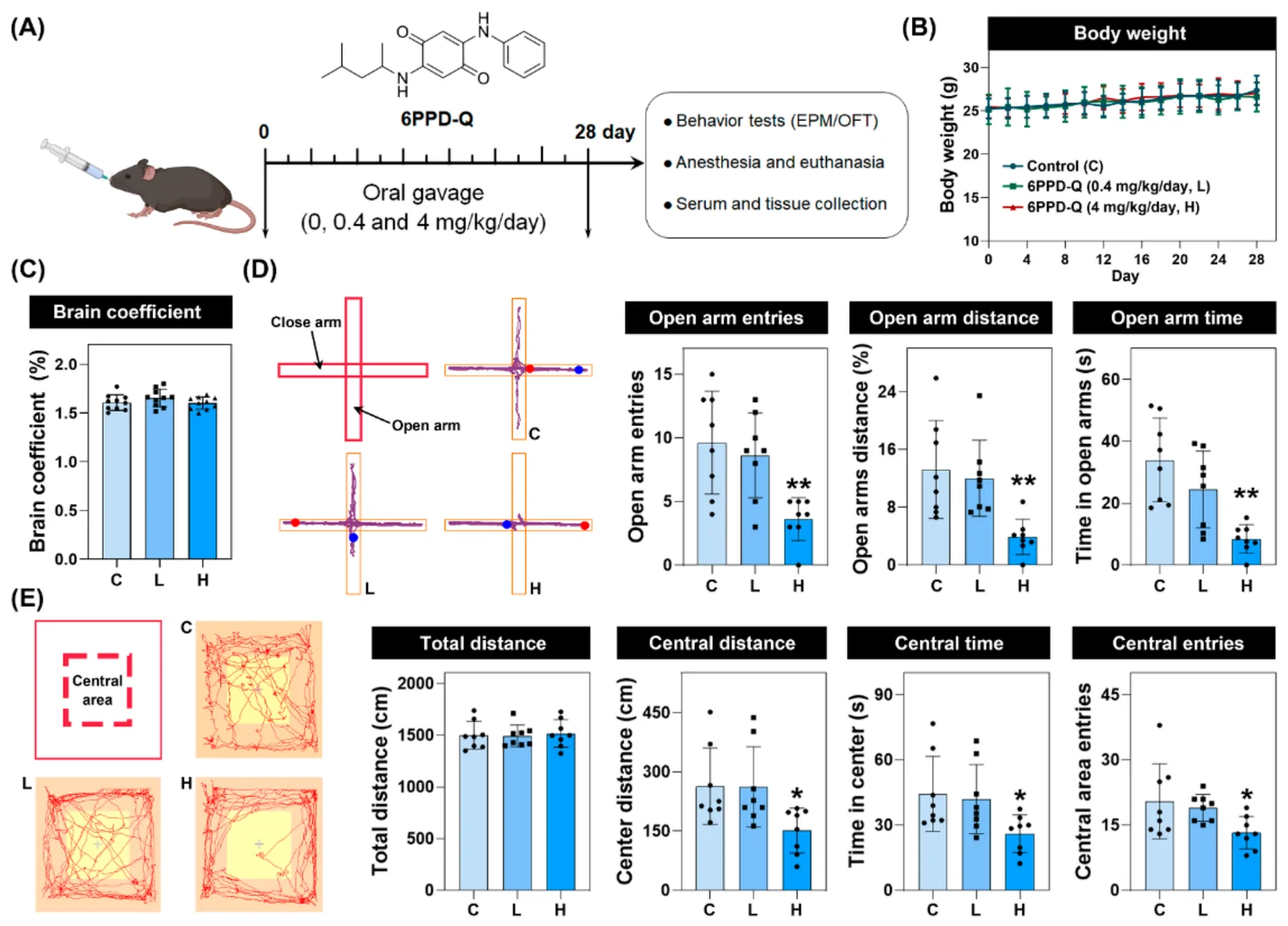
Establishment of 6PPD-Q-induced anxiety-like behavior in mice. (**A**) Schematic of the experimental design for 6PPD-Q exposure. (**B**) Body weight and (**C**) brain organ coefficient of mice across experimental groups (*n =* 10). (**D**) Behavioral assessment in the EPM test: representative movement traces, open arm entries, distance traveled in open arms, and time spent in open arms (*n =* 8). (**E**) Behavioral assessment in the OFT: representative movement traces, total distance traveled, distance in center, time in center, and entries into center (*n* = 8). Data are expressed as mean ± SD. * *p* < 0.05, ** *p* < 0.01, compared with the control group.

Two mouse exposure models were established in this study (Figure 1A). In the first model, thirty mice were weight-randomized into three groups (*n* = 10) and administered daily gavage for 28 consecutive days. The groups consisted of a vehicle control (olive oil, C) and two treatment groups receiving 0.4 (low-dose, L) or 4 (high-dose, H) mg/kg/day of 6PPD-Q in olive oil. The doses were selected based on previous studies, taking into account 6PPD-Q’s bioaccumulative potential and trophic magnification to ensure detectable effects without mortality [27,28]. In the second model, forty mice were weight-randomized into four groups (*n =* 10) and received daily gavage for 28 days, consistent with the first model. The groups were: (1) vehicle control (C), (2) 6PPD-Q (4 mg/kg/day, H), (3) the agonist CGS 21680 (2 mg/kg via intraperitoneal injection every 4 days, CG), and (4) a combination of 6PPD-Q and CGS 21680 (H + CG). The dosage of CGS 21680 was selected based on a previous study, and CGS 21680 was administered 2 h prior to each 6PPD-Q treatment [29]. In the first animal model, 10 mice per group were randomly housed in two cages (5 mice/cage), resulting in three groups, 30 mice, and six cages. In the second model, 10 mice per group were similarly housed in two cages (5 mice/cage), resulting in four groups, 40 mice, and eight cages. Cage positions were randomly assigned to minimize potential environmental effects. Throughout the study, all mice were monitored daily and weighed every two days. At the endpoint, animals underwent behavioral tests and were then euthanized under anesthesia for the collection of brain tissue and blood.

### 2.3. Elevated Plus Maze (EPM) Test and Open Field Test (OFT)

The EPM test was performed on day 28 of 6PPD-Q exposure to evaluate anxiety-like behavior in mice. The apparatus consisted of two open arms (30 × 6 cm) and two closed arms (30 × 6 cm, with 15-cm high walls) connected by a central platform (6 × 6 cm). Each mouse was placed on the central platform facing an open arm, and its 5-min exploratory activity was recorded using a digital camera. The number of entries into, time spent in, and distance traveled in each arm were analyzed using SuperMaze V2.0 software.

The OFT was performed after the EPM test to evaluate spontaneous locomotor activity and exploratory behavior of mice in a novel environment. The apparatus consisted of a plastic chamber (40 × 40 cm) featuring white flooring and interior walls. Testing was performed under low illumination in a quiet room. At the start of the test, each mouse was placed gently in the center of the arena, facing the same direction. A 5-min recording of the animal’s movement was captured using a digital camera. The chamber was cleaned with 75% ethanol and aired for 2 min between trials. Behavioral parameters, including time spent in the central area, number of central entries, and total distance traveled were analyzed using the EthoVision XT 17.0 animal motion tracking system. Behavioral data with obvious recording or testing abnormalities were excluded from the analysis.

### 2.4. Measurement of 6PPD-Q in Serum and Brain

The concentrations of 6PPD-Q in serum and brain tissues were determined by ultra-performance liquid chromatography-tandem mass spectrometry (UPLC-MS/MS; AB Sciex, Framingham, MA, USA) following the previously established method [30,31]. Briefly, serum (100 μL) and cerebral tissue (100 mg) samples were processed for chemical extraction. Serum was mixed with three volumes of ice-cold methanol, while tissue was homogenized in 500 μL of acetonitrile using steel beads. Both sample types were then vortexed for 1 min and centrifuged at 12,000× *g* for 15 min at 4 °C. The resulting supernatants were collected, dried under a nitrogen stream, and the residues were reconstituted in 100 μL of methanol/water (1:1, *v*/*v*). For analysis, chromatographic separation of the 10 μL injected sample was achieved on an ACQUITY UPLC BEH C18 column (maintained at 40 °C) using a mobile phase composed of 0.1% formic acid in water (A) and acetonitrile (B), delivered at a flow rate of 0.3 mL/min. Mass spectrometric detection was performed in positive ESI-MRM mode. The optimized instrument parameters were as follows: both 6PPD-Q and the deuterated internal standard 6PPD-Q-D5 eluted at a retention time of 2.7 min. For 6PPD-Q, the precursor ion was monitored at *m*/*z* 299.1, and product ions were detected at *m*/*z* 187.1 (quantifier), 200, and 241.1. The corresponding cone voltages were set to 25 V for each transition, with collision energies of 20, 20, and 15 eV, respectively. For 6PPD-Q-D5, the precursor ion was recorded at *m*/*z* 304.1, and the product ions were *m*/*z* 192.1 (quantifier), 205, and 246.1. Cone voltages of 30, 25, and 25 V were applied, together with collision energies of 30, 40, and 30 eV for the respective transitions.

### 2.5. Transmission Electron Microscopy (TEM) Detection

After extraction, the cerebral tissues were immediately cooled on ice. A coronal block (2–3 mm thick) containing the amygdala was precisely dissected under the guidance of a brain atlas. The samples were then fixed in 2.5% glutaraldehyde at 4 °C for 24 h in the dark. This was followed by post-fixation in 1% osmium tetroxide, dehydration through a graded ethanol series (50–100%), and embedding in resin. Ultrathin sections were obtained and double-stained with uranyl acetate and lead citrate. The ultrastructure of the blood-brain barrier (BBB) in the amygdala was then examined using a TEM (HT7800, HITACHI, Tokyo, Japan).

### 2.6. Histopathological Examination

After perfusion, cerebral tissues were fixed in 4% paraformaldehyde for 24 h, followed by dehydration, paraffin embedding, and microtomy to generate 5-μm sections. These sections were stained with hematoxylin and eosin (H&E) or Nissl solution as per the manufacturer’s instructions. Whole-slide images were acquired using either an OLYMPUS OlyVIA system (Olympus Corporation, Tokyo, Japan) or a Pannoramic MIDI scanner (3DHISTECH, Budapest, Hungary).

### 2.7. Golgi–Cox Staining

Cerebral tissues were immediately isolated and immersed in freshly prepared impregnation solutions for two weeks. Following cryoprotection at 4 °C for one week, the tissues were frozen in liquid nitrogen and sectioned at 50 µm using a cryostat under conditions of −20 °C to −22 °C. The resulting sections were mounted on gelatin-coated slides, air-dried for 72 h at room temperature, and stained as per the manufacturer’s protocol. Whole-slide images were acquired with a Pannoramic MIDI scanner (3DHISTECH, Budapest, Hungary).

### 2.8. Fluoro-Jade B (FJB) Staining

The paraffin-embedded cerebral sections were deparaffinized and hydrated through a series of immersions: 1% sodium hydroxide in 80% alcohol (5 min), 70% alcohol (2 min), and distilled water (2 min). The sections were then treated with 0.06% potassium permanganate, rinsed, and incubated with FJB solution for 30 min. After rinsing and drying, the sections were counterstained with DAPI and imaged using a fluorescence microscope (Carl Zeiss, Jena, Germany).

### 2.9. Immunofluorescence Assay

The paraffin-embedded cerebral sections were deparaffinized and subjected to antigen retrieval by heating in citrate buffer. After blocking with serum from the primary antibody host species (1 h, room temperature), the sections were sequentially incubated with primary antibodies overnight at 4 °C and fluorescent secondary antibodies at 37 °C for 1 h. Following this, the sections were rinsed, counterstained with DAPI, and scanned using a fluorescence whole slide scanner (KF-FL-020, Jiangfeng, China).

### 2.10. Transcriptome Sequencing and Analysis

Total RNA was extracted from the dissected amygdala-containing samples using Trizol reagent and assessed for purity and integrity. Sequencing libraries were constructed by BioMarker (Beijing, China) with the Hieff NGS Ultima Dualmode mRNA Library Prep Kit and sequenced on an Illumina NovaSeq 6000 platform. After quality control, the high-quality clean reads were aligned to the reference genome using Hisat2. Differential expression analysis was performed with DESeq2, applying thresholds of |fold change| > 2.0 and an adjusted *p*-value < 0.01. Functional enrichment of the identified genes was subsequently profiled using the Kyoto Encyclopedia of Genes and Genomes (KEGG) and Gene Ontology (GO) databases.

### 2.11. Quantitative Real-Time PCR (qRT-PCR)

Confirmation of the transcriptomic results was conducted by qRT-PCR on selected genes. Following the RNA extraction protocol described above, cDNA was synthesized using the HiScript IV All-in-one RT SuperMix. Gene-specific primers, designed and synthesized by Generay, were used with the ChamQ Universal SYBR qPCR Master Mix on an Applied Biosystems 7500 system. The thermal cycling protocol comprised an initial denaturation at 95 °C for 30 s, followed by 40 cycles of 95 °C for 10 s and 60 °C for 30 s. Gene expression levels were quantified relative to the endogenous control β-actin using the 2^−ΔΔCt^ method. Primer sequences for each gene are listed in Table A2.

### 2.12. Network Toxicology Analysis

Ten PPD-Qs were selected for network toxicology and subsequent molecular docking analyses. Of these, the SMILES structures of seven (6PPD-Q, 7PPD-Q, 77PPD-Q, CPPD-Q, IPPD-Q, DPPD-Q, and DTPD-Q) were retrieved from the PubChem database (https://pubchem.ncbi.nlm.nih.gov/ (accessed on 17 March 2026)), while those of the remaining three (8PPD-Q, 44PD-Q, and 66PD-Q) were derived from a previous study [31]. Potential protein targets of these compounds were identified by screening three independent databases: ChEMBL (https://www.ebi.ac.uk/chembl/ (accessed on 17 March 2026)), Swiss Target Prediction (http://www.swisstargetprediction.ch/ (accessed on 17 March 2026)), and TargetNet (https://targetnet.scbdd.com/calcnet/index/ (accessed on 17 March 2026)). Targets associated with “Anxiety-like behavior” (D001008) were obtained from the Comparative Toxicogenomics Database (https://ctdbase.org/ (accessed on 17 March 2026)). An intersection analysis was then performed to identify common targets between the PPD-Q-associated and anxiety disorder-related targets, visualized by a Venn diagram. Functional enrichment of the identified targets was subsequently profiled using the KEGG and GO databases.

### 2.13. Molecular Docking

Molecular docking analysis was performed to explore the binding interactions between PPD-Qs and cAMP-modulating proteins from human and mouse, including dopamine receptors (DRD1-DRD5), neuropeptide receptors (GHRH and NPS), adrenergic receptors (ADRA2A and ADRA2C, ADRB1, and ADRB2), GABA-B receptor (GABBR1), adenosine receptors (ADORA1, ADORA2A, and ADORA2B), PDEs (PDE4A, PDE4C and PDE4D), and serotonin receptors (HTR4, HTR6, HTR7, and HTR1B). Due to the limited availability of experimental co-crystal structures, we employed AlphaFold-predicted models for all proteins, using their UniProtKB accession numbers. Molecular simulations were conducted using Schrödinger Maestro 12.8. The protein structure was prepared with the Protein Preparation Wizard module, involving hydrogen addition, optimization of hydrogen bonds, removal of waters beyond 3 Å, and hydrogen minimization using the OPLS4 force field. A docking grid was generated with the Receptor Grid Generation module, centered on the ligand-binding domain coordinates with a dimension of 36 Å to encompass all potential binding sites. The 3D structures of PPD-Qs were constructed and energy-minimized with the LigPrep module under the OPLS4 force field. Molecular docking was performed using the XP (extra precision) mode with flexible ligand sampling, and the pose with the highest docking score was selected for further analysis. In addition, for a more accurate molecular docking analysis between 6PPD-Q and its key target ADORA2A, we supplemented the computational models with the protein’s reported crystal structure retrieved from the Protein Data Bank (PDB: 3QAK).

### 2.14. Molecular Dynamics Simulations

Molecular dynamics simulations were performed using GROMACS 2022. The system was parameterized with the pdb2gmx tool and the AutoFF web server, applying the CHARMM36 force field to the receptor and CGenFF to the ligand. The system was then solvated in a cubic box with TIP3P water molecules, maintaining a 1-nm distance between the solute and the box boundary, and neutralized with ions using the gmx genion tool. Non-bonded interactions were calculated with a 1-nm cutoff, while long-range electrostatics were treated using the Particle Mesh Ewald (PME) method. All bonds were constrained with the SHAKE algorithm, and integration was performed with a 1-fs time step via the Verlet leap-frog algorithm. Energy minimization was conducted sequentially: 3000 steps of the steepest descent method followed by 2000 steps of the conjugate gradient method, progressively releasing constraints on the solute and counterions. Subsequently, a 50-ns production simulation was carried out under NPT conditions at 310 K. Trajectory analysis included the calculation of the root-mean-square deviation (RMSD), root-mean-square fluctuation (RMSF), number of hydrogen bonds (H-bonds), radius of gyration (Rg), solvent-accessible surface area (SASA), and Gibbs free energy using the corresponding GROMACS tools (gmx rms, gmx rmsf, gmx hbond, gmx gyrate, gmx sasa, and gmx sham or gmx bar).

### 2.15. Cell Culture and Treatment

The mouse neuroblastoma cell line Neuro-2a (N2a) was purchased from the Cell Bank of the Chinese Academy of Sciences (Shanghai, China). Cells were cultured in MEM supplemented with 10% FBS and 1% penicillin-streptomycin at 37 °C in a humidified 5% CO_2_ incubator. To evaluate the cytotoxicity of 6PPD-Q, cells were seeded in 96-well plates at a density of 4 × 104 cells per well. After adherence, the cells were treated with a concentration gradient of 6PPD-Q (0–335 μM) for 24 h, with six replicate wells per concentration. Cell viability was assessed using a standard CCK-8 assay. For rescue experiments, cells were pretreated with the selective ADORA2A agonist CGS 21680 (10 μM) for 1 h prior to 6PPD-Q exposure.

### 2.16. Measurement of cAMP in the Mouse Amygdala and N2a Cells

The levels of cAMP in the dissected amygdala-containing samples and N2a cells were quantified using a commercial ELISA kit, strictly following the manufacturer’s instructions. The absorbance for each sample was measured at 450 nm.

### 2.17. Western Blot Analysis

Proteins were extracted from the dissected amygdala-containing samples and N2a cells with RIPA lysis buffer containing protease and phosphatase inhibitors, and their concentrations were quantified using a BCA assay. The resulting lysates represented total protein extracts. The lysates were subsequently denatured at 100 °C for 5 min, and equal amounts of total protein were separated by 10% SDS-PAGE and electrophoretically transferred onto polyvinylidene difluoride (PVDF) membranes. After blocking with 5% skim milk in TBST for 1 h at room temperature, the membranes were incubated with primary antibodies overnight at 4 °C. Following washes with TBST, the membranes were incubated with HRP-conjugated secondary antibodies for 1 h at room temperature. The protein bands were finally visualized using a chemiluminescence detection system and quantified densitometrically with ImageJ 1.54g software.

### 2.18. Statistical Analysis

Data are presented as mean ± SD. Statistical analyses were performed using SPSS 17.0. The distribution of the data was assessed before statistical analysis. For datasets with sufficient sample sizes, normality was assessed using the Shapiro–Wilk test. Because formal normality testing is not considered reliable for datasets with very small sample sizes (e.g., *n* = 3), normality was not formally tested for these datasets. Differences between two groups were assessed using an independent-samples t-test, while multi-group comparisons were evaluated using one-way or two-way ANOVA followed by Fisher’s LSD test. For repeated body-weight measurements, two-way repeated-measures ANOVA was used to assess changes over time and between treatment groups. A *p*-value < 0.05 was considered statistically significant. For histological, immunofluorescence, and morphological analyses, n represents the number of independent animals. One section was obtained from each animal, and one or two fields were randomly selected from each section for observation and measurement. Measurements from multiple fields within the same animal were averaged, and the resulting animal-level means were used for statistical analysis. Thus, repeated measurements from the same animal were not treated as independent samples.

## 3. Results

### 3.1. Establishment of 6PPD-Q-Induced Anxiety-like Behavior in Mice

Throughout the exposure experiment (Figure 1A), mice in all three groups exhibited comparable body weight gain and no abnormalities in general health or appearance (Figure 1B). Furthermore, the relative weight of the brain was unaffected by 6PPD-Q treatment (Figure 1C), indicating that this compound was well-tolerated at the tested concentrations, with no evidence of systemic toxicity.

The established behavioral assays, such as the EPM and OFT, provide reliable metrics to quantify anxiety-like behavior in mice. In the EPM, high-dose 6PPD-Q exposure induced a pronounced preference for the closed arms, as evidenced by significantly fewer open-arm entries and reduced distance traveled and time spent in the open arms (Figure 1D). Consistent with this anxiogenic phenotype, 6PPD-Q also significantly reduced exploratory behavior in the OFT, decreasing the central distance, number of entries, and time spent in the center area (Figure 1E). Importantly, although 6PPD-Q altered exploratory behavior in the EPM and OFT, the total travel distance in the OFT was comparable across groups, providing no evidence of a generalized reduction in locomotor activity. Nevertheless, the potential contribution of altered locomotor or exploratory activity to the interpretation of anxiety-like behavior cannot be completely excluded. No significant differences were observed between the low-dose and the control group on any of these measures. Collectively, these results demonstrate that high-dose exposure to 6PPD-Q induces robust anxiety-like behavior in mice.

### 3.2. 6PPD-Q Permeates the Brain and Elicits Direct Histopathological Damage in the Mouse Amygdala

**Figure 2:**
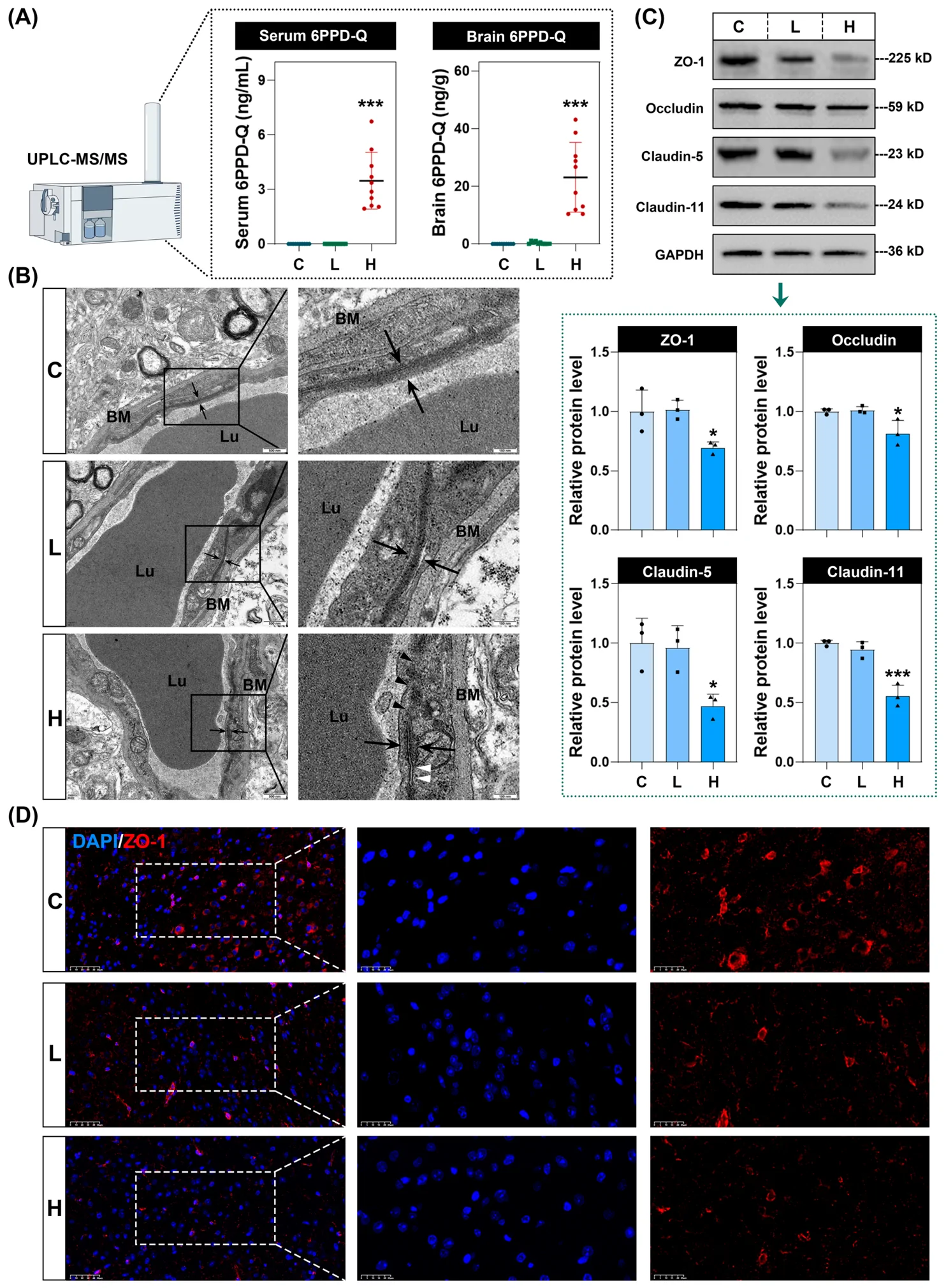
6PPD-Q penetrates the brain following BBB disruption. (**A**) UPLC-MS/MS analysis of 6PPD-Q concentrations in mouse serum and brain tissue (*n* = 10). (**B**) Representative TEM images of the mouse BBB. BM, basement membrane; Lu, lumen; black arrows, intact TJs; black triangles, fragmented TJs; white triangles, faded TJs. Scale bars: 500 nm (left), 150 nm (right). (**C**) Western blot analysis of tight junction proteins (ZO-1, occludin, claudin-5, claudin-11) in the mouse amygdala (*n* = 3). Band intensities were quantified using ImageJ. (**D**) Representative immunoflu-orescence images of ZO-1 (red) and DAPI (blue) in the mouse amygdala. Scale bars: 50 μm (left), 25 μm (right, magnification). Data are expressed as mean ± SD. * *p* < 0.05, *** *p* < 0.001, compared with the control group.

Given that the BBB serves to prevent circulating toxicants from entering the central nervous system (CNS) [32,33], we next investigated whether 6PPD-Q can enter the brain to exert neurotoxic effects or induce anxiety-like behavior indirectly. UPLC-MS/MS quantification revealed significant 6PPD-Q accumulation in brain tissue, especially in the high-dose group (Figure 2A). Ultrastructurally, high-dose 6PPD-Q disrupted the defined BBB architecture observed in controls, causing tight junction (TJ) fragmentation, disruption, and disorganization (Figure 2B). Consistent with these morphological changes, expression of key TJ proteins (ZO-1, Occludin, Claudin-5, and Claudin-11) was significantly reduced (Figure 2C), and immunofluorescence analysis confirmed a marked decrease in ZO-1 localization within the amygdala of treated mice (Figure 2D). Collectively, these findings demonstrate that 6PPD-Q compromises BBB integrity by disrupting TJ complexes, a mechanism that likely facilitates its brain entry.

**Figure 3:**
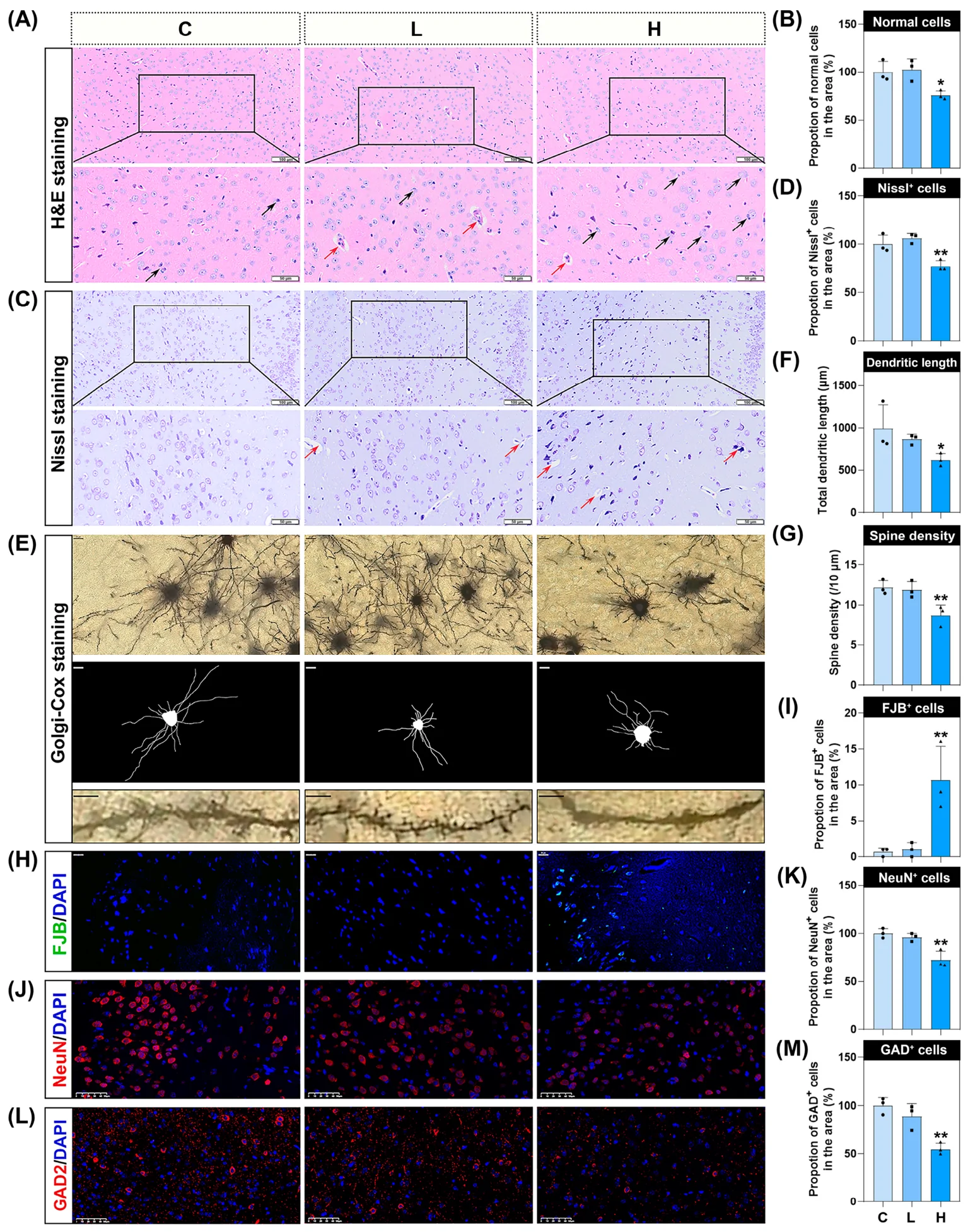
The induction of neuronal damage in the amygdala of mice following 6PPD-Q exposure. (**A**,**B**) H&E staining of the amygdala and quantification of normal neurons. (**C**,**D**) Nissl staining of the amygdala and quantification of Nissl+ bodies. Black arrows indicate deformation of neurons; Red arrows indicate edema of neurons. Scale bars: 100 μm (top panel); 50 μm (bottom panel). (**E**–**G**) Golgi–Cox staining of dendritic spines in the amygdala (**E**) and quantification of total dendritic branch length (**F**) and dendritic spine density (**G**). Scale bars: 20 μm (upper two panels), 5 μm (bottom panel). (**H**,**I**) FJB staining of degenerating neurons (**H**) and quantification of FJB+ cells (**I**). Scale bar: 20 μm. (**J**,**K**) NeuN immunofluorescence staining (**J**) and quantification of NeuN+ neurons (**K**). Scale bar: 50 μm. (**L**,**M**) GAD2 immunofluorescence staining (**L**) and quantification of GAD2+ neurons (**M**). Scale bars: 50 μm. Data are expressed as mean ± SD (*n* = 3). * *p* < 0.05, ** *p* < 0.01, compared with the control group.

As the amygdala serves as a central hub in the neural network governing anxiety-like behaviors [34], we subsequently assessed the deleterious impact of 6PPD-Q on amygdalar neurons. Histological examination (H&E and Nissl staining) demonstrated that the normal, well-organized neuronal architecture in controls was severely compromised by 6PPD-Q treatment, which induced neuronal deformation, edema and disarray, and a reduction in Nissl bodies, leading to a substantial loss of normal neurons (Figure 3A–D). Furthermore, Golgi–Cox staining revealed a pronounced impairment in neuronal connectivity, with significant reductions observed in both total dendritic length and spine density (Figure 3E–G). This evidence of neuronal loss was further supported by immunofluorescence staining, which showed a significant reduction in NeuN-positive neurons in the amygdala of 6PPD-Q-treated mice compared to the controls (Figure 3J,K). Consistently, FJB staining showed a marked increase in FJB-positive degenerating neurons (Figure 3H,I) following 6PPD-Q exposure, confirming ongoing neuronal loss and degeneration. Notably, this neurodegenerative profile was accompanied by a marked decrease in GAD2 immunofluorescence intensity in the amygdala (Figure 3L,M). Collectively, these data indicate that 6PPD-Q induces profound neurodegenerative changes and GABAergic deficits in the amygdala, which are associated with the observed anxiety-like behaviors.

### 3.3. cAMP Pathway Suppression Is a Potential Mechanism Associated with 6PPD-Q-Driven Anxiety-like Behavior, with Similar Patterns Predicted Across PPD-Qs

**Figure 4:**
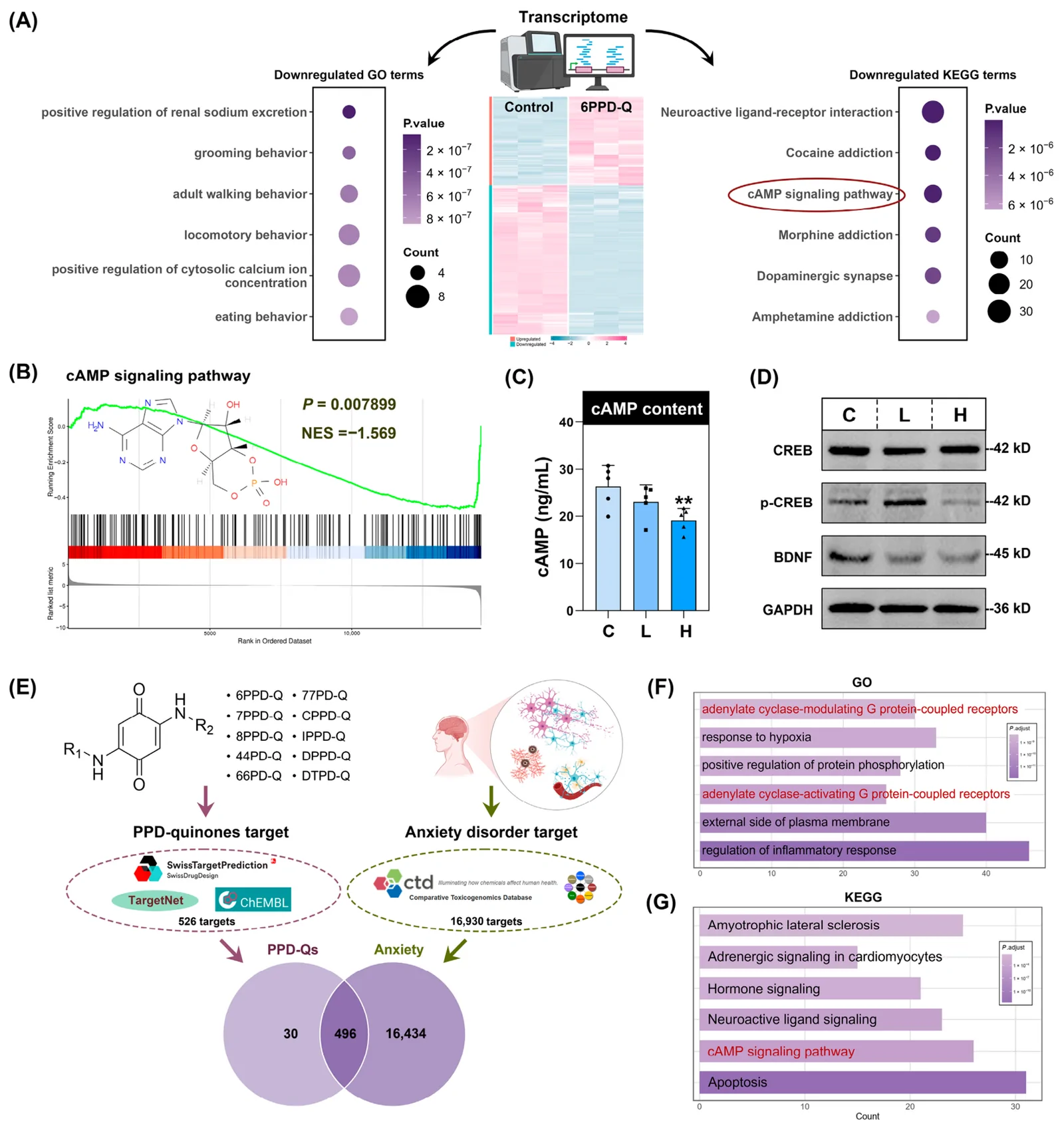
cAMP pathway suppression is a potential mechanism associated with 6PPD-Q-driven anxiety-like behavior, with similar patterns observed across PPD-Qs. (**A**) Heatmap depicting the downregulated DEGs and their enrichment in repre-sentative GO terms and KEGG pathways. (**B**) GSEA showing significant negative en-richment of the cAMP signaling pathway in the 6PPD-Q group. (**C**) Quantification of cAMP in the mouse amygdala (*n* = 5). (**D**) Western blot analysis of cAMP signaling proteins (CREB, p-CREB, BDNF) in the mouse amygdala (*n* = 3). (**E**) Venn diagram illustrating the overlap between predicted targets of PPD-Qs and anxiety disorder-associated genes. (**F**,**G**) GO enrichment analysis (**F**) and KEGG pathway analysis (**G**) of the 496 overlapping targets implicated in PPD-Q-related anxiety-like behavior. Data are expressed as mean ± SD. ** *p* < 0.01, compared with the control group.

**Figure A1:**
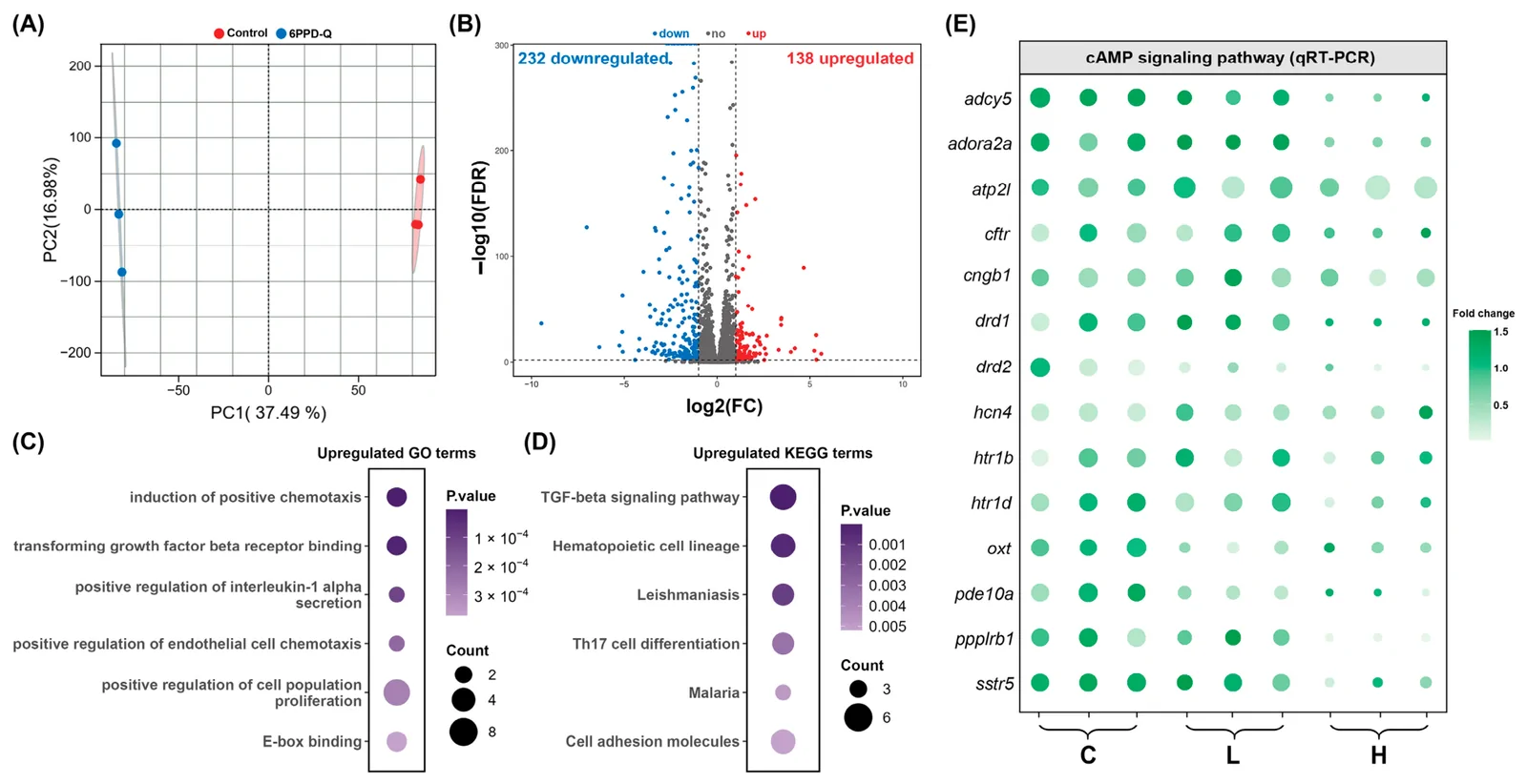
Transcriptomic analysis of the mouse amygdala. (**A**) Principal component analysis (PCA) of amygdala transcriptomes from control and 6PPD-Q-treated mice (*n* = 3). (**B**) Volcano plot of differentially expressed genes (DEGs). (**C**,**D**) Enrichment analysis of upregulated DEGs for (**C**) GO and (**D**) KEGG pathways. (**E**) Validation of DEGs related to cAMP signaling pathway in the amygdala (*n* = 3). The mRNA expression levels of target genes were quantified by qRT-PCR using gene-specific primers. *β-actin* served as an internal control.

**Figure A2:**
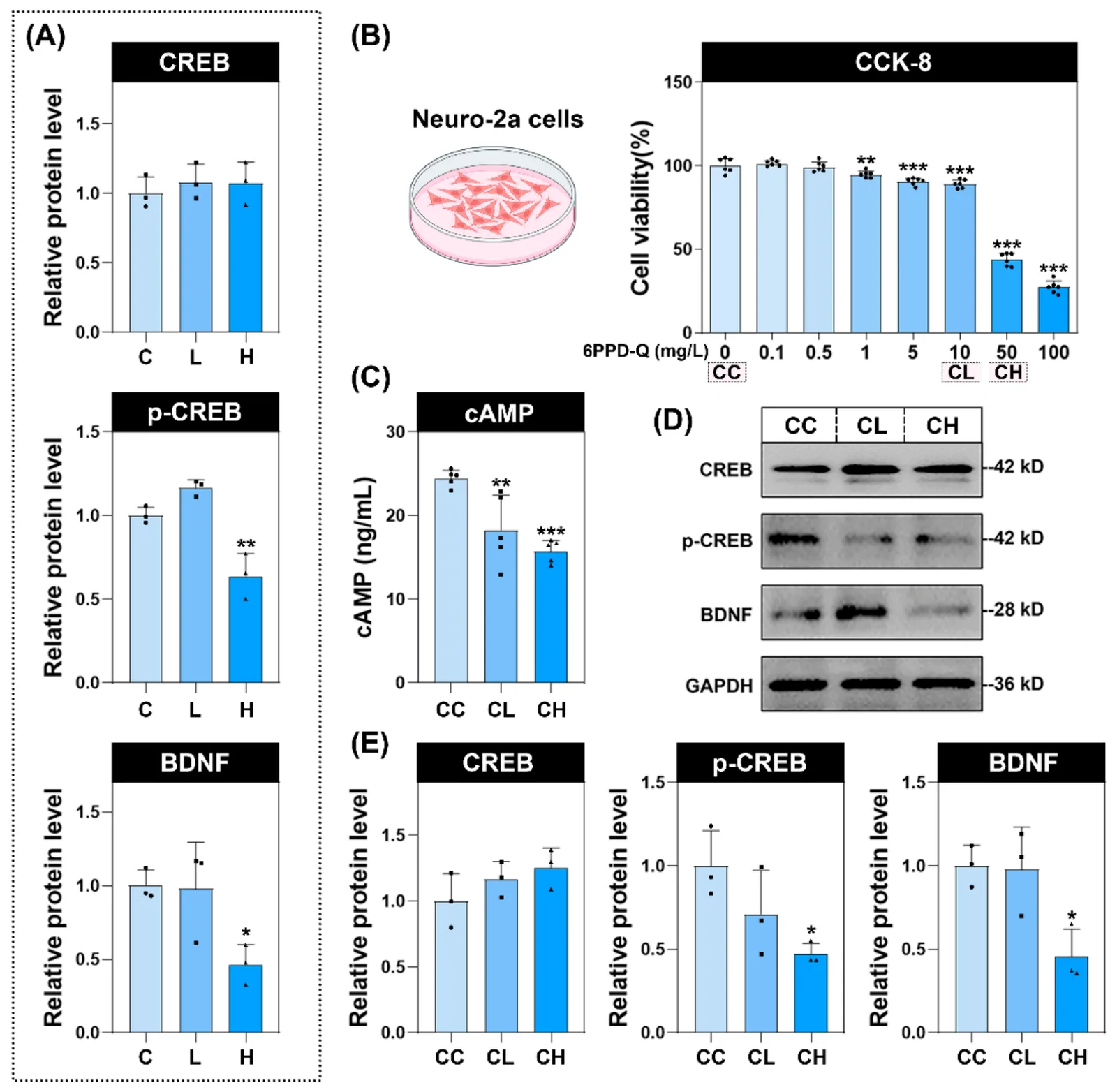
The inhibition of the cAMP signaling pathway in the mouse amygdala and N2a cells following 6PPD-Q exposure. (**A**) Quantitative analysis of cAMP signaling protein expression in the mouse amygdala (*n* = 3). (**B**) Effects of 6PPD-Q on cytotoxicity in N2a cells. Cell viability and representative images of N2a cells exposed to different concentrations of 6PPD-Q (*n* = 6). (**C**) Quantification of cAMP in N2a cells (*n* = 5). (**D**,**E**) Western blot analysis of cAMP signaling proteins in N2a cells (*n* = 3). Data are expressed as mean ± SD. * *p* < 0.05, ** *p* < 0.01, *** *p* < 0.001, compared with the control group.

To further elucidate the mechanisms by which 6PPD-Q induces amygdala impairment and anxiety-like behavior, transcriptomic analysis was performed on amygdala tissue from control and 6PPD-Q-treated mice. Principal component analysis (PCA) revealed distinct transcriptomic profiles between the two groups (Figure A1A). Specifically, we identified 370 differentially expressed genes (DEGs), comprising 138 upregulated and 232 downregulated genes (Figure A1B). GO and KEGG analyses indicated that 6PPD-Q-induced downregulation predominantly affected genes involved in behavioral processes, neuroactive ligand-receptor interaction, and the cAMP signaling pathway (Figure 4A), whereas upregulated genes were mainly associated with the TGF-beta signaling pathway and neuroinflammation (Figure A1C,D). Subsequent Gene Set Enrichment Analysis (GSEA) revealed a significant negative enrichment of the cAMP signaling pathway (Figure 4B), a finding that was further validated by qRT-PCR analysis of key pathway components (Figure A1D). As a result, 6PPD-Q significantly suppressed cAMP production and downregulated the protein levels of p-CREB and BDNF in the mouse brain (Figure 4C,D and Figure A2A) and N2a cells (Figure A2B–E). These findings demonstrate that 6PPD-Q exposure suppresses the cAMP/CREB/BDNF signaling cascade, a molecular alteration that is associated with the observed neurobehavioral abnormalities and amygdalar damage, and may contribute to 6PPD-Q neurotoxicity.

To determine whether similar molecular patterns are observed across other PPD-Qs, we next performed a network toxicology analysis to identify potential targets linking PPD-Qs exposure to anxiety-like behavior. As shown in Figure 4E, we identified 526 potential molecular targets common to the ten PPD-Qs examined (6PPD-Q, 7PPD-Q, 8PPD-Q, 44PD-Q, 66PD-Q, 77PD-Q, CPPD-Q, IPPD-Q, DPPD-Q, and DTPD-Q) from the SwissTargetPrediction, TargetNet, and ChEMBL databases. Concurrently, 16930 anxiety disorder-associated genes were retrieved from the Comparative Toxicogenomics Database. Intersection analysis revealed 496 overlapping candidates shared between PPD-Qs and anxiety-like behavior (Figure 4E). GO enrichment analysis of the overlapping targets demonstrated their significant association with AC-related GPCRs, which are intimately associated with cAMP production (Figure 4F). Consistently, KEGG pathway analysis revealed that these overlapping targets were significantly enriched in the cAMP signaling pathway (Figure 4G). Taken together, these findings indicate that cAMP signaling-related targets and pathways were commonly identified across the tested PPD-Qs in the computational analyses.

### 3.4. ADORA2A Is a Potential Target Associated with cAMP Inhibition by 6PPD-Q

**Figure 5:**
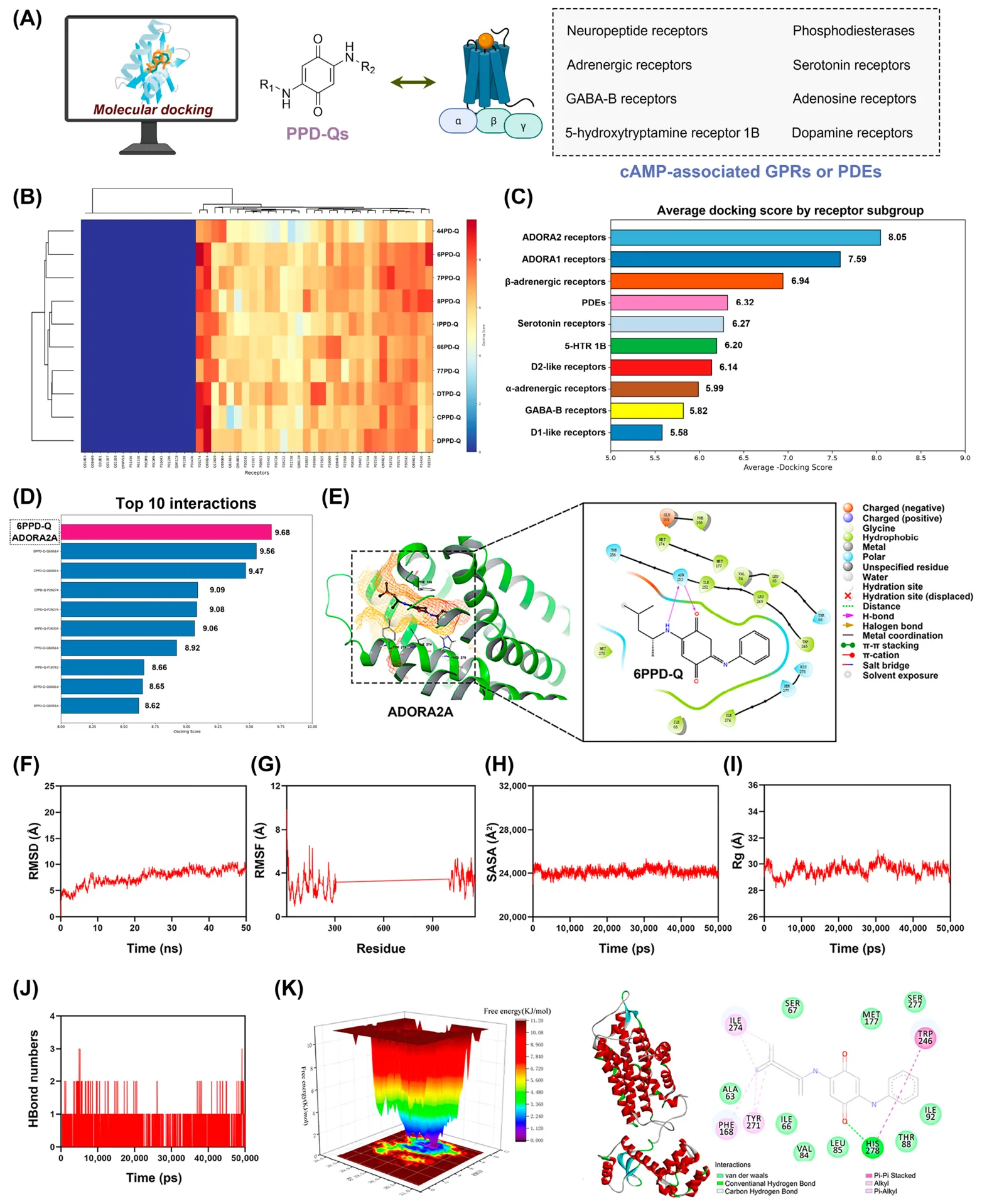
Predicted interaction between 6PPD-Q and ADORA2A and its potential association with cAMP inhibition. (**A**) Molecular docking analysis of the binding interactions between PPD-Qs and cAMP-modulating proteins from human and mouse, including dopamine receptors, neuropeptide receptors, adrenergic receptors, GABA-B receptor, adenosine receptors, PDEs, and serotonin receptors. (**B**) Heatmap showing the docking scores of PPD-Qs with cAMP-modulating proteins. (**C**) Average docking scores of PPD-Qs with cAMP-modulating proteins by subfamily. (**D**) Top 10 binding interactions between PPD-Qs and cAMP-modulating proteins. (**E**) Three-dimensional molecular docking pose of 6PPD-Q bound to ADORA2A. (F-K) Molecular dynamics simulations of the 6PPD-Q–ADORA2A complex, showing RMSD (**F**), RMSF (**G**), SASA (**H**), Rg (**I**), number of hydrogen bonds (**J**), and FEL (**K**).

Given that intracellular cAMP signaling is tightly controlled by GPCRs and PDEs [35], we further employed molecular docking to evaluate the potential interactions between PPD-Qs and these critical cAMP-modulating proteins, including neuropeptide receptors, adrenergic receptors, serotonin receptors, GABA-B receptors, adenosine receptors, dopamine receptors, and PDEs (Figure 5A). The heatmap depicts the docking scores of PPD-Qs with these proteins (Figure 5B). Furthermore, the ADORA2 subgroup exhibited the highest average docking score for PPD-Qs (Figure 5C). Analysis of the top 10 predicted binding interactions revealed that 6PPD-Q displayed the highest docking score with ADORA2A, followed by DPPD-Q and CPPD-Q, both with ADORA2B (Figure 5D). As the predicted 6PPD-Q–ADORA2A interaction exhibited the most favorable docking score not only among all evaluated PPD-Qs but also across all protein targets examined, we selected this complex as a representative case for detailed structural analysis. Three-dimensional molecular docking showed that the aromatic ring of 6PPD-Q was positioned within the ADORA2A orthosteric binding pocket and formed a hydrogen bond with ASN253, a site essential for receptor-ligand interactions (Figure 5E) [36].

**Figure A3:**
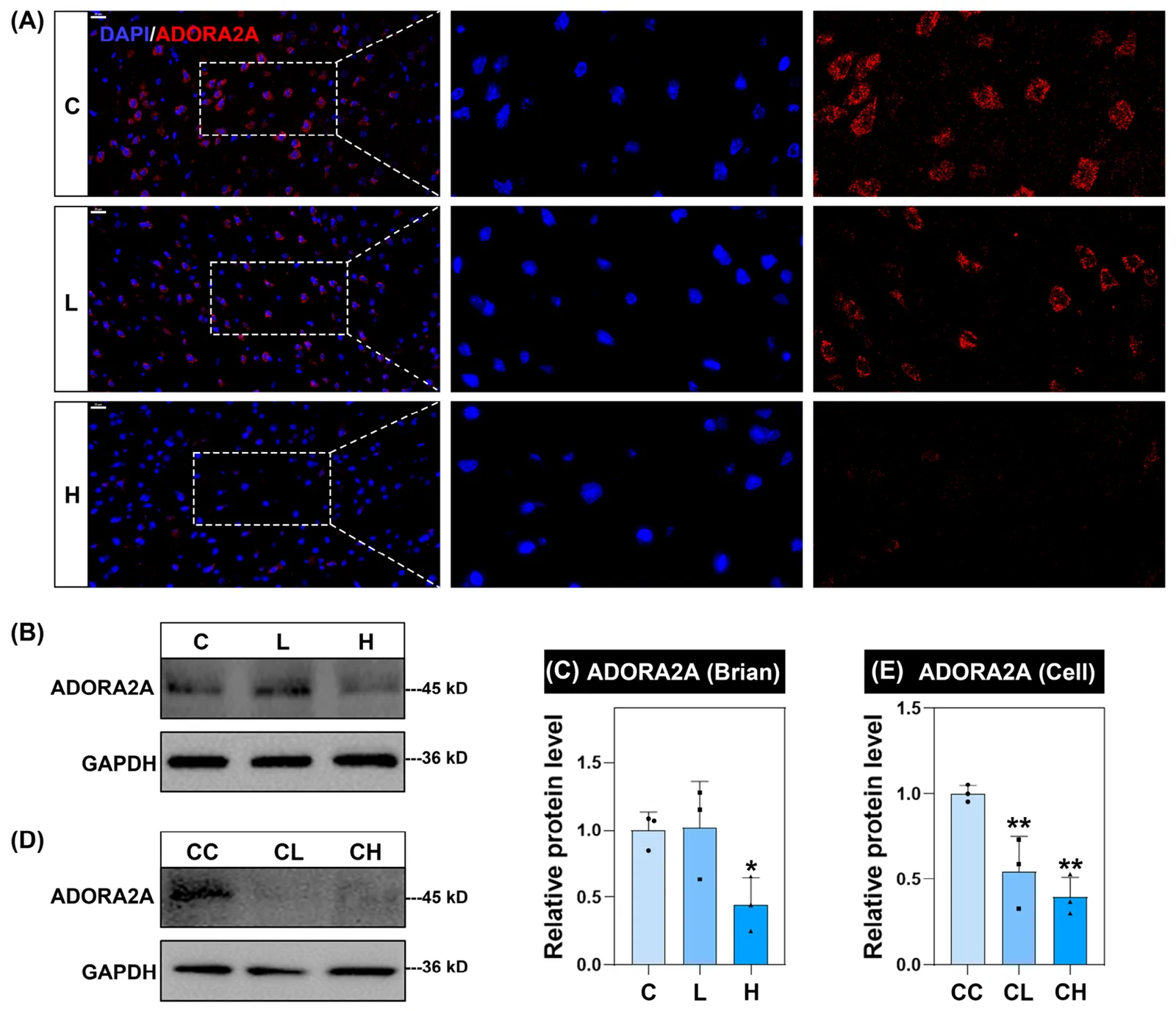
6PPD-Q decreases the expression of ADORA2A in the mouse amygdala and N2a cells. (**A**) Representative images of fluorescence staining for ADORA2A (red) and DAPI (blue) in the mouse amygdala. Scale bars: 20 μm. (**B**–**E**) Western blot analysis of ADORA2A protein expression in (**B**,**C**) the mouse amygdala and (**D**,**E**) N2a cells (*n* = 3). Data are expressed as mean ± SD. * *p* < 0.05, ** *p* < 0.01, compared with the control group.

We next performed molecular dynamics simulations to further characterize the predicted 6PPD-Q–ADORA2A binding mode. While the overall protein conformation remained stable (Figure 5F–H), 6PPD-Q shifted deeper into the binding pocket (Figure 5I). This movement brought HIS278 into proximity with the quinone ring, enabling a new hydrogen bond (Figure 5J,K). Concurrently, a previously solvent-exposed side chain of the ligand penetrated deeper, engaging TYR271, PHE168, and ILE274 in hydrophobic interactions (Figure 5K), supporting the stability of the predicted 6PPD-Q–ADORA2A interaction. Notably, these interacting residues are highly conserved within the ADORA2A orthosteric binding sites [36,37,38]. To further investigate the potential involvement of ADORA2A, we also examined ADORA2A protein levels in 6PPD-Q-treated mice and N2a cells. We found that 6PPD-Q significantly decreased the expression of ADORA2A in both the mouse amygdala and N2a cells (Figure A3). Collectively, these results support ADORA2A as a potential target involved in 6PPD-Q-associated cAMP signaling changes, based on computationally predicted interactions and changes in ADORA2A protein levels.

### 3.5. Pharmacological Activation of ADORA2A with CGS 21680 Alleviates 6PPD-Q-Induced Neurotoxicity and Anxiety-like Behaviors

**Figure 6:**
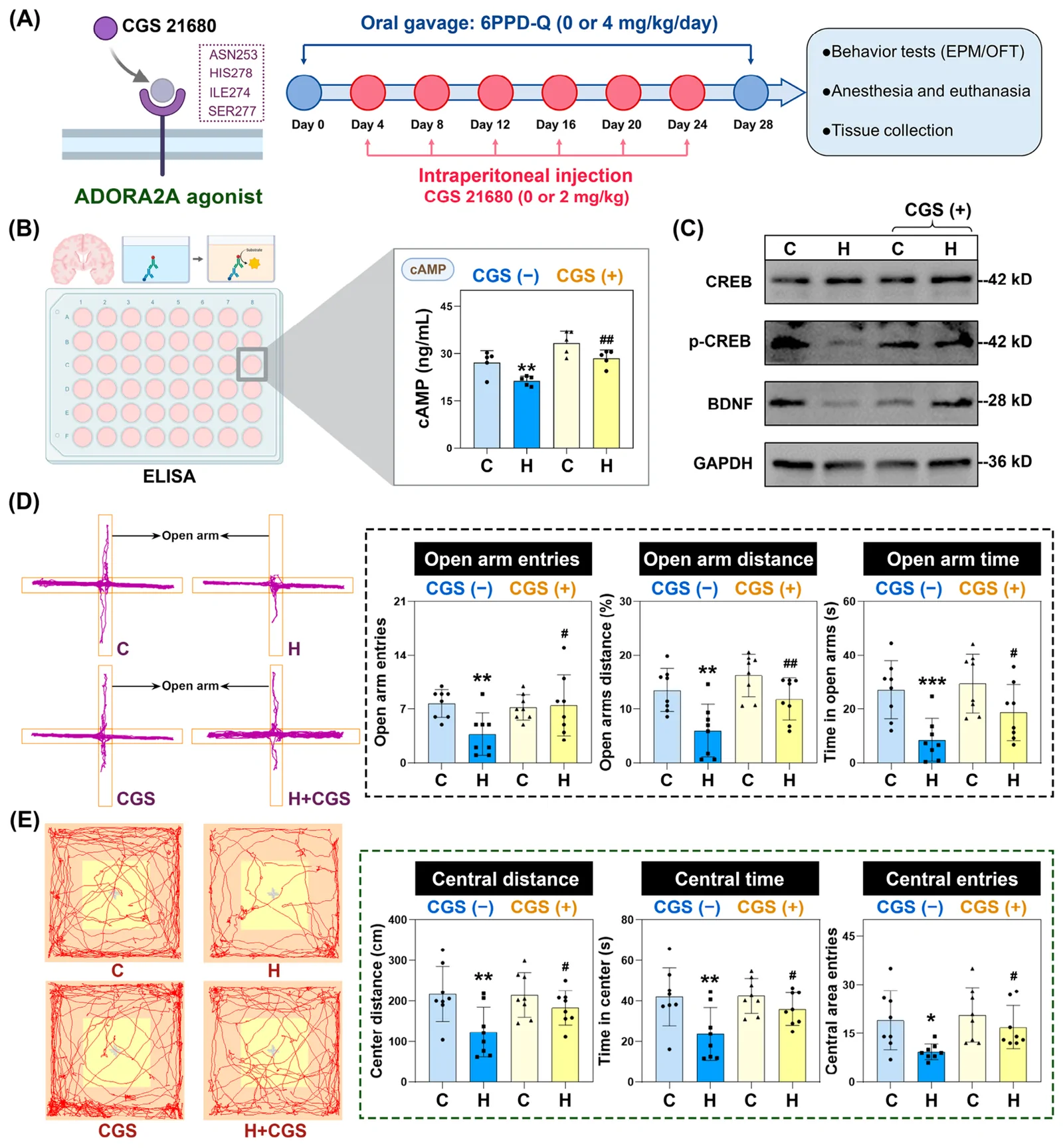
Pharmacological activation of ADORA2A alleviates 6PPD-Q-induced cAMP inhibition and anxiety-like behaviors. (**A**) Schematic of experimental design for 6PPD-Q exposure and CGS 21680 administration. (**B**) Quantification of cAMP in the mouse amygdala (*n* = 5). (**C**) Western blot analysis of cAMP signaling proteins in the mouse amygdala (*n* = 3). (**D**) Behavioral assessment in the EPM test: representative movement traces, open arm entries, distance traveled in open arms, and time spent in open arms (*n* = 8). (**E**) Behavioral assessment in the OFT: representative movement traces, total distance traveled, distance in center, time in center, and entries into center (*n* = 8). * *p* < 0.05, ** *p* < 0.01, *** *p* < 0.001, compared with the control group; # *p* < 0.05, ## *p* < 0.01, compared with the 6PPD-Q group.

**Figure A4:**
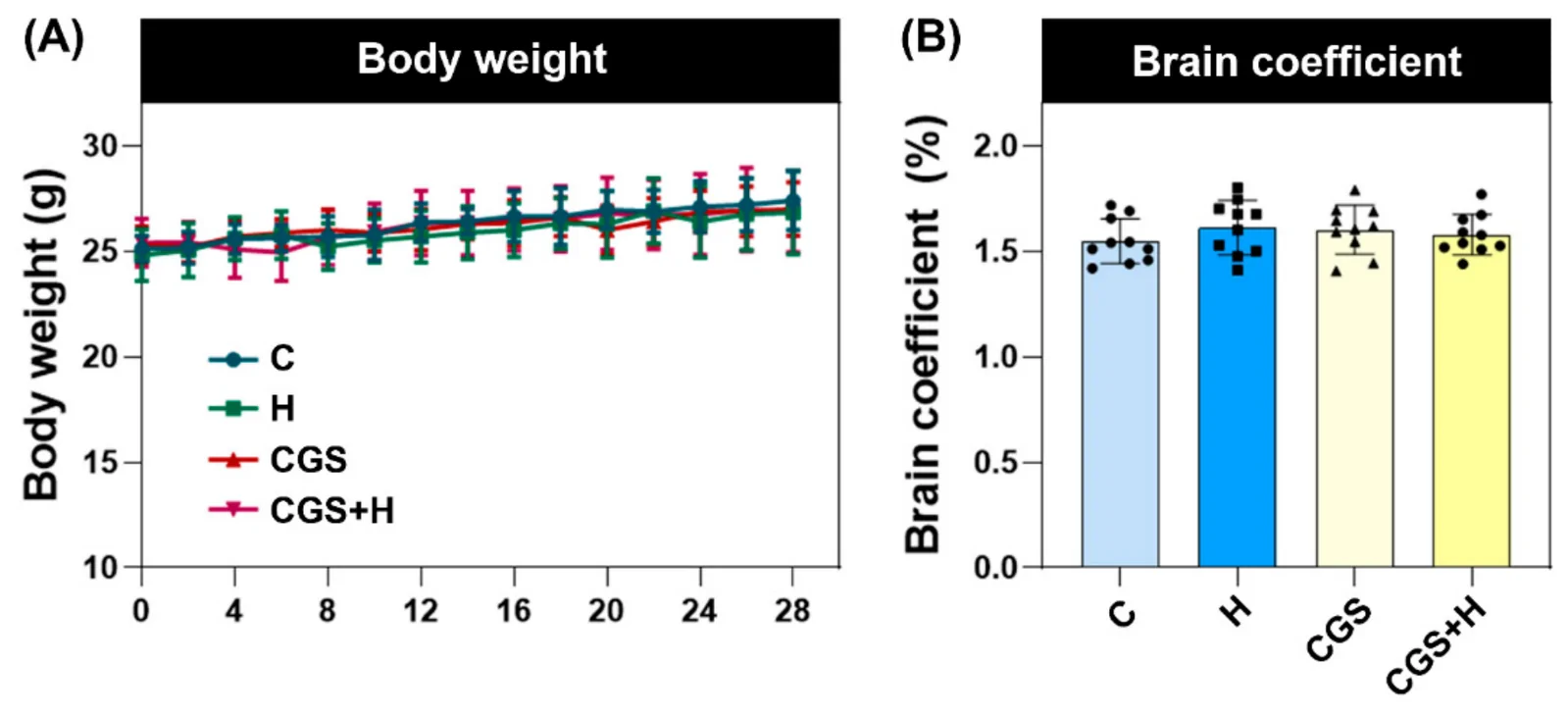
Body weight and relative brain weight in mice treated with 6PPD-Q and CGS 21680. (**A**) Body weight and (**B**) brain organ coefficient of mice across experimental groups (*n* = 10). Data are expressed as mean ± SD.

**Figure A5:**
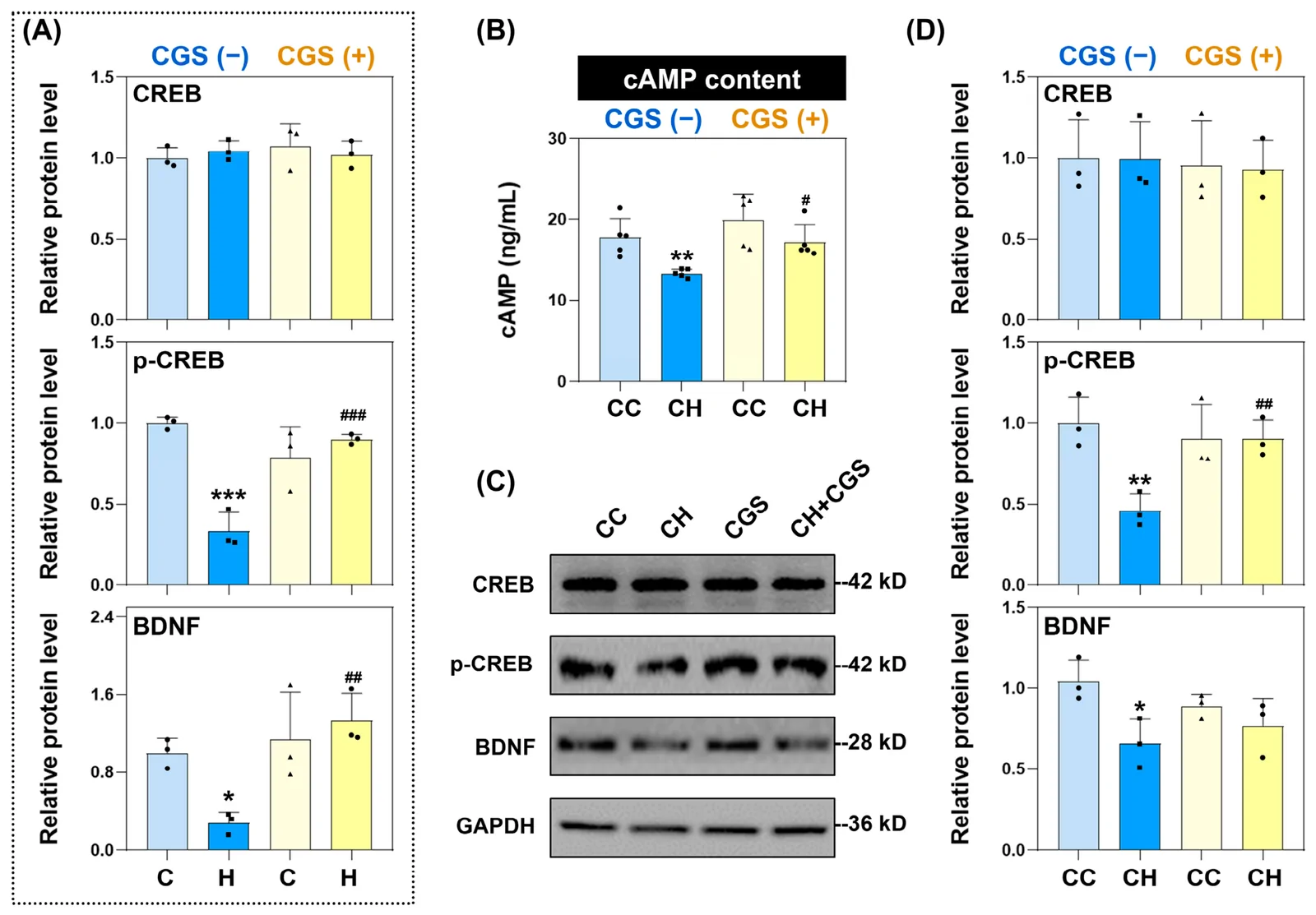
Activation of ADORA2A reverses 6PPD-Q-induced suppression of cAMP signaling in the mouse amygdala and N2a cells. (**A**) Quantitative analysis of cAMP signaling protein expression in the mouse amygdala (*n* = 3). (**B**) Quantification of cAMP in N2a cells (*n* = 5). (**C**,**D**) Western blot analysis of cAMP signaling proteins in N2a cells (*n* = 3). Data are expressed as mean ± SD. * *p* < 0.05, ** *p* < 0.01, *** *p* < 0.001, compared with the control group; ^#^ *p* < 0.05, ^##^ *p* < 0.01, ^###^ *p* < 0.001, compared with the 6PPD-Q group.

CGS 21680 is a selective ADORA2A agonist that interacts with ADORA2A at HIS278, ILE274, and ASN253, residues that are also predicted to interact with 6PPD-Q [39,40,41]. To further investigate the involvement of ADORA2A in 6PPD-Q-associated cAMP suppression and neuronal impairment, we performed an in vivo rescue experiment using CGS 21680 (Figure 6A). The results showed that neither CGS 21680 nor 6PPD-Q significantly altered mouse body weight or the relative weight of the brain (Figure A4). Moreover, compared to the 6PPD-Q group, CGS 21680 co-treatment significantly restored cAMP levels and increased the expression of p-CREB and BDNF in the mouse amygdala (Figure 6B,C and Figure A5A) and N2a cells (Figure A5B–D). Additionally, the abnormal behavioral changes in the 6PPD-Q-exposed mice were significantly alleviated by CGS 21680. As shown in Figure 6D,E, CGS 21680 treatment significantly reversed the behavioral deficits induced by 6PPD-Q exposure in both the EPM and OFT tests, specifically the reductions in entries, distance traveled, and time spent in the open arms and center area, respectively.

**Figure 7:**
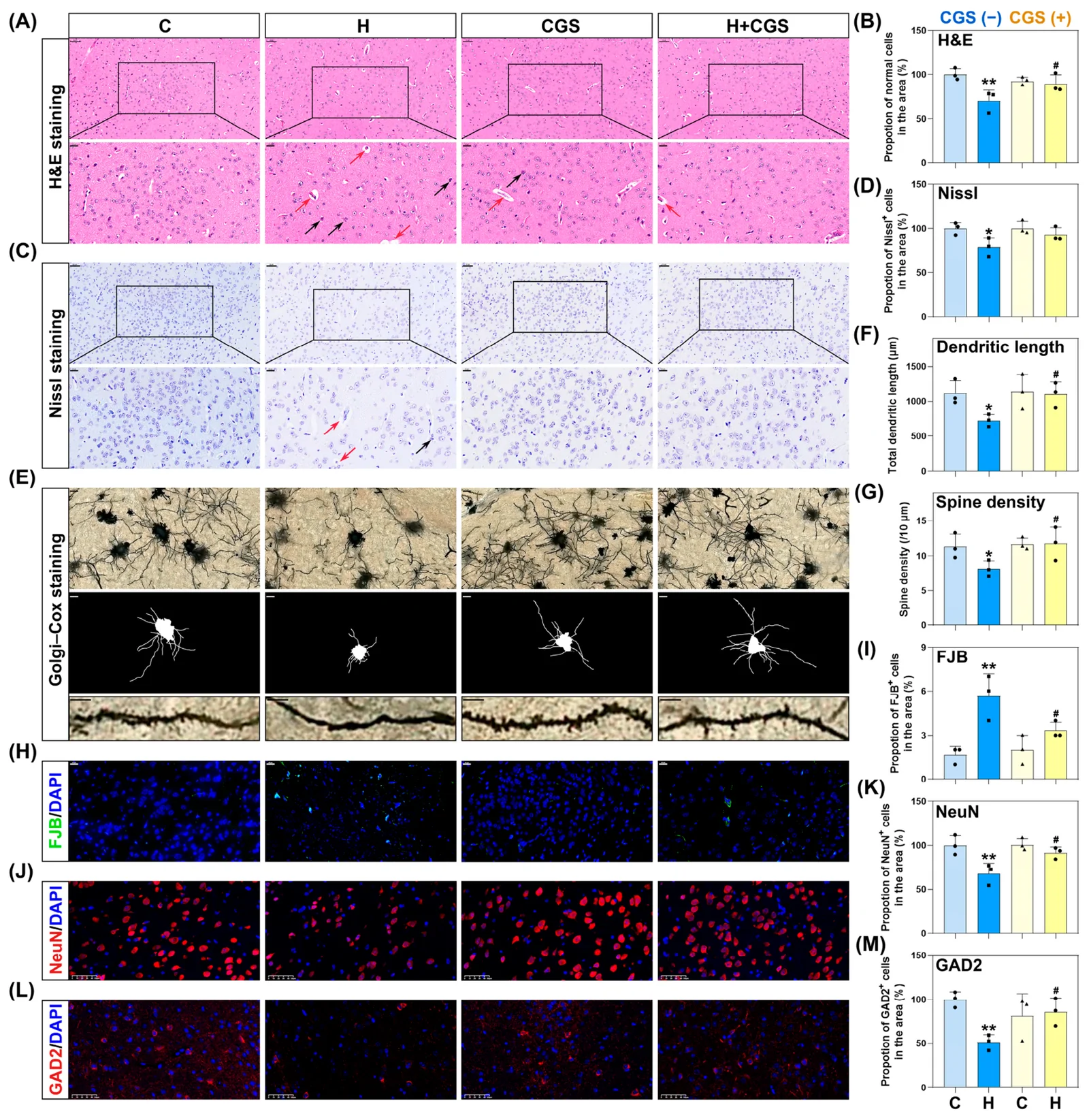
Activation of ADORA2A alleviates 6PPD-Q-induced amygdalar damage. (**A**,**B**) H&E staining of the amygdala and quantification of normal neurons. (**C**,**D**) Nissl staining of the amygdala and quantification of Nissl+ bodies. Black arrows indicate deformation of neurons; Red arrows indicate edema of neurons. Scale bars: 100 μm (top panel); 50 μm (bottom panel). (**E**–**G**) Golgi–Cox staining of dendritic spines in the amygdala (**E**) and quantification of total dendritic branch length (**F**) and dendritic spine density (**G**). Scale bars: 20 μm (upper two panels), 5 μm (bottom panel). (**H**,**I**) FJB staining of degenerating neurons (**H**) and quantification of FJB+ cells (**I**). Scale bar: 20 μm. (**J**,**K**) NeuN immunofluorescence staining (**J**) and quantification of NeuN+ neurons (**K**). Scale bar: 50 μm. (**L**,**M**) GAD2 immunofluorescence staining (**L**) and quantification of GAD2+ neurons (**M**). Scale bars: 50 μm. Data are expressed as mean ± SD (*n* = 3). * *p* < 0.05, ** *p* < 0.01, compared with the control group, # *p* < 0.05, compared with the 6PPD-Q group.

The neuroprotective effects of CGS 21680 against 6PPD-Q toxicity were also evidenced by H&E and Nissl staining, which showed restored neuronal morphology and increased numbers of normal neurons and Nissl vesicles in the mouse amygdala (Figure 7A–D). Concurrently, quantitative morphometric analysis revealed that both the total dendritic length and spine density in the amygdala were significantly greater in mice treated with 6PPD-Q + CGS 21680 than in those treated with 6PPD-Q alone (Figure 7E–G). Moreover, CGS 21680 supplementation concomitantly reduced the number of degenerated neurons (indicated by FJB staining) and increased the population of NeuN-positive neurons in the mouse amygdala (Figure 7H–K). The 6PPD-Q-induced decrease in amygdalar GAD2 fluorescent intensity was also rescued by CGS 21680 treatment (Figure 7L,M). Taken together, these results indicate that pharmacological activation of ADORA2A by CGS 21680 alleviates 6PPD-Q-induced cAMP suppression and associated neurotoxicity, supporting the involvement of ADORA2A in these effects.

## 4. Discussion

Global mental health is fundamental to human well-being and societal productivity. Among the spectrum of mental disorders, anxiety conditions have emerged as a leading contributor to disability and diminished quality of life, with their rising incidence increasingly attributed to environmental exposures [42,43]. This trend underscores the urgent need to elucidate the environmental drivers of anxiety pathogenesis. In this work, we demonstrated that 6PPD-Q exposure compromises the BBB, allowing the compound to permeate the CNS and induce damage to amygdalar neurons, thereby eliciting anxiety-like behaviors in mice. We further found that suppression of the cAMP signaling cascade is associated with 6PPD-Q-induced anxiety, with molecular docking and molecular dynamics simulations suggesting a potential interaction between 6PPD-Q and the orthosteric ligand-binding pocket of ADORA2A [36,37,38]. Moreover, computational analyses suggested similar ADORA2A-associated patterns among other PPD-Qs, although these predictions have not been experimentally validated. These findings offer a novel mechanistic framework for understanding the neurotoxicity of 6PPD-Q and provide evidence for a potential molecular axis associated with PPD-Q-associated health risks.

It is well recognized that environmental toxicants can induce neurotoxicity through direct or indirect mechanisms, depending on their capacity to penetrate the BBB, a specialized endothelial interface that restricts the entry of circulating xenobiotics into the CNS, thereby protecting neural tissue from potential insult [33,44]. Thus, determining whether a pollutant can cross the BBB and accumulate in the CNS is a critical first step in assessing its neurotoxic potential. In this study, by using an experimental model of anxiety-like behavior in mice, we detected the compound in both serum and brain tissues of exposed animals, confirming its systemic absorption and brain penetration and thereby establishing a critical foundation for evaluating its neurotoxic implications. Of note, the serum 6PPD-Q levels detected in mice from the high-dose group (1.95 to 6.75 ng/mL) were comparable to those reported in outdoor workers from Chongqing, China (0.58 to 4.04 ng/mL) [45]. This observation indicates that the exposure levels used in this study are within the range of reported human exposure levels, although differences in exposure kinetics, tissue distribution, metabolism, and biological responses should be considered when interpreting the potential human relevance of these findings. Moreover, we also found that exposed mice exhibited disrupted BBB ultrastructure, reduced expression of TJ proteins, and overt damage to amygdalar neurons. These findings support the occurrence of neurotoxic effects following 6PPD-Q exposure and brain penetration.

The amygdala plays an essential role in the regulation of anxiety [46]. Notably, while acute and extensive damage to the amygdala, whether from targeted lesions, stroke or surgery, can disrupt excitatory signal generation and reduce threat responsiveness, the underlying mechanisms differ under chronic conditions such as physical stress or environmental pollutant exposure [47]. In these conditions, anxiety is associated with amygdalar neuronal degeneration and functional impairment [48]. For instance, the anxiety-like behavior induced by chronic restraint stress was mediated by structural impairment in the mouse amygdala, which was characterized by reduced dendritic spine density and an aberrant shift in spine morphology [49]. Moreover, aspartame consumption promoted anxiety in mice via amygdalar downregulation of the GABA-A receptor associated protein, leading to a shift in the E/I balance toward excitation [50]. Supporting this, our results confirmed neuronal damage in the amygdala following 6PPD-Q exposure, including structural deformation, edema, and loss of GAD2-positive neurons, NeuN-positive mature neurons, and Nissl bodies. This damage may directly impair dendrites and their dendritic spines, which are critical structures for receiving and integrating synaptic inputs [51]. As expected, Golgi–Cox staining demonstrated that 6PPD-Q exposure led to a significant reduction in both dendritic spine density and total branching length within the mouse amygdala. These data support the presence of amygdalar neuropathological changes associated with 6PPD-Q-induced anxiety-like behavior and set the stage for investigating upstream mechanisms.

Through integrative transcriptomic, molecular and biochemical analyses, we identified that reduced cAMP levels and the suppression of the cAMP/CREB/BDNF signaling axis were associated with 6PPD-Q-induced anxiety-like behavior in mice. This finding is consistent with previous reports implicating the cAMP/CREB/BDNF cascade in anxiety-like behaviors elicited by traditional neurotoxicants [21,52]. Moreover, given the structural and chemical similarities among PPD-Qs, we next investigated whether 6PPD-Q-induced cAMP suppression reflects a class-wide mechanism. Network toxicology analysis of ten representative PPD-Qs revealed that the overlapping targets between these pollutants and anxiety-like behavior are significantly enriched in AC-coupled GPCRs and cAMP signaling cascade. These computational findings indicate that cAMP signaling-related targets and pathways are commonly represented across the tested PPD-Qs, providing a basis for investigating whether similar mechanisms may occur across this chemical class. Additionally, although cAMP is a well-established second messenger whose downstream signaling cascades have been extensively characterized in the context of anxiety and neuronal function [53,54], how environmental pollutants interface with its regulatory machinery to initiate these disruptions remains poorly understood. This raises a fundamental question: what is the potential molecular target through which PPD-Qs may interface with the cAMP pathway?

To address this, we employed molecular docking analysis to screen for potential cAMP-regulatory proteins that may interact with PPD-Qs, including stimulatory Gs-coupled receptors, inhibitory Gi-coupled receptors, and degrading PDEs. Across the tested PPD-Qs, the ADORA2 subfamily ranked highest in average docking score, with the 6PPD-Q–ADORA2A pair achieving the most favorable predicted interaction among all combinations examined. Additionally, the stability of the 6PPD-Q–ADORA2A predicted complex was supported by RMSD and RMSF, key metrics for conformational stability where lower values indicate a more stable complex [55]. Although the complex underwent conformational changes throughout its motion, as shown by Rg fluctuations, the free energy landscape (FEL) of the complex showed that 6PPD-Q exhibited a tendency to penetrate deeper into the ADORA2A receptor. This inward movement was accompanied by additional contacts with key receptor residues, supporting the stability of the predicted interaction. Thus, ADORA2A emerges as a prominent potential molecular target through which PPD-Qs, exemplified by 6PPD-Q, may interface with the cAMP signaling cascade. ADORA2A, a Gs-coupled adenosine receptor with high ligand affinity, is predominantly localized to presynaptic terminals of the brain, where it modulates neuronal excitability, neurotransmitter release, and synaptic plasticity [56,57]. Upon activation, ADORA2A couples to Gs proteins to stimulate AC, thereby elevating intracellular cAMP, engaging the PKA-CREB signaling axis, and functioning as a critical regulator of anxiety-like behaviors [58,59].

Moreover, molecular docking and molecular dynamics simulations predicted that 6PPD-Q can interact with the orthosteric ligand-binding pocket of ADORA2A, involving key residues including ASN253, HIS278, TYR271, PHE168, and ILE274. This predicted binding mode raises the possibility that 6PPD-Q may interfere with adenosine receptor activation, thereby contributing to downstream cAMP suppression. To further investigate the potential involvement of ADORA2A, we employed CGS 21680, a well-characterized ADORA2A agonist that has been reported to interact with residues including HIS278, ILE274, and ASN253 [37,39,40,41], Pharmacological activation of ADORA2A with CGS 21680 restored cAMP levels, reactivated the CREB-BDNF axis, and ameliorated amygdalar neuronal damage and anxiety-like behaviors in 6PPD-Q-exposed mice. These findings provide pharmacological evidence supporting the involvement of ADORA2A in 6PPD-Q-induced cAMP suppression and associated neurobehavioral effects. In addition, given that ADORA2A is localized to the neuronal plasma membrane [60], the predicted interaction between 6PPD-Q and its orthosteric pocket may represent a potential molecular initiating event (MIE) within the adverse outcome pathway (AOP) framework, although this interpretation remains to be experimentally validated. Further studies are required to determine whether 6PPD-Q directly binds ADORA2A, characterize its binding affinity and receptor occupancy, and establish whether it functionally antagonizes ADORA2A.

However, several limitations should be acknowledged. Although each treatment group was distributed across two cages, the statistical analyses did not explicitly account for potential within-cage correlation. Therefore, cage-related effects cannot be completely excluded. Future studies should include more independent cages per treatment group and apply hierarchical or mixed-effects models to better account for cage-level effects. In addition, although a 24-h interval was provided between the EPM and OFT to minimize potential carry-over effects, the influence of the preceding EPM on subsequent OFT performance cannot be completely excluded. Future studies could consider counterbalancing the order of behavioral tests or using independent cohorts to further minimize potential order effects. Finally, the molecular docking and molecular dynamics analyses provide a structural basis for a potential 6PPD-Q–ADORA2A interaction but cannot establish direct receptor binding, binding affinity, receptor occupancy, or functional antagonism. In addition, although changes in cAMP signaling, CREB/BDNF signaling, amygdalar pathology, and anxiety-like behaviors were observed in association with 6PPD-Q exposure, the present study did not experimentally establish a sequential causal relationship among these molecular, pathological, and behavioral events. Direct binding and receptor functional assays and pathway-specific interventions will therefore be required to determine whether ADORA2A represents a molecular initiating event and whether cAMP/CREB/BDNF signaling causally contributes to the observed neurobehavioral effects in PPD-Q-induced neurotoxicity.

## 5. Conclusions

This study provides evidence that 6PPD-Q exposure is associated with suppression of the cAMP/CREB/BDNF signaling axis, amygdalar neuronal damage, and anxiety-like behaviors in mice, with molecular docking and molecular dynamics simulations predicting a potential interaction between 6PPD-Q and the orthosteric ligand-binding pocket of ADORA2A. Computational analyses further identified similar ADORA2A- and cAMP-related patterns across the tested PPD-Q congeners, although these findings remain to be experimentally validated. Notably, pharmacological activation of ADORA2A with CGS 21680 alleviated the molecular, pathological, and behavioral effects associated with 6PPD-Q exposure, providing pharmacological evidence supporting the involvement of ADORA2A. Collectively, these findings provide a potential mechanistic framework linking PPD-Q exposure to anxiety-like behavioral effects and highlight ADORA2A-associated cAMP signaling as a candidate pathway for further investigation. Direct receptor-binding and functional studies are warranted to determine whether the predicted 6PPD-Q–ADORA2A interaction represents a bona fide molecular initiating event and whether similar ADORA2A-associated mechanisms occur across other PPD-Q congeners.

## Data Availability

The raw data supporting the conclusions of this article will be made available by the authors on request.

## Abbreviations

The following abbreviations are used in this manuscript:

6PPD-Q	6-(N-phenyl-p-phenylenediamine) quinone
PPD	p-Phenylenediamine
PPD-Qs	p-Phenylenediamine quinones
cAMP	Cyclic adenosine monophosphate
BDNF	Brain-derived neurotrophic factor
GPCRs	G protein-coupled receptors
AC	Adenylyl cyclase
PDEs	Phosphodiesterases
ADORA2A	Adenosine A2A receptor
BBB	Blood-brain barrier
CNS	Central nervous system
SPF	Specific pathogen-free
EPM	Elevated plus maze
OFT	Open field test
UPLC-MS/MS	Ultra-performance liquid chromatography-tandem mass spectrometry
TEM	Transmission electron microscopy
H&E	Hematoxylin and eosin
FJB	Fluoro-Jade B
DAPI	4′,6-Diamidino-2-phenylindole
qRT-PCR	Quantitative real-time polymerase chain reaction
PCA	Principal component analysis
DEGs	Differentially expressed genes
GO	Gene Ontology
KEGG	Kyoto Encyclopedia of Genes and Genomes
GSEA	Gene Set Enrichment Analysis
PDB	Protein Data Bank
RMSD	Root-mean-square deviation
RMSF	Root-mean-square fluctuation
Rg	Radius of gyration
SASA	Solvent-accessible surface area
FEL	Free energy landscape
MIE	Molecular initiating event
AOP	Adverse outcome pathway
N2a	Neuro-2a
MEM	Minimum Essential Medium
FBS	Fetal bovine serum
DMSO	Dimethyl sulfoxide
ELISA	Enzyme-linked immunosorbent assay
BCA	Bicinchoninic acid
SDS-PAGE	Sodium dodecyl sulfate-polyacrylamide gel electrophoresis
TBST	Tris-buffered saline with Tween 20
ANOVA	Analysis of variance
LSD	Least significant difference
SD	Standard deviation

## Appendix A

**Table A1:** The primary antibodies used in this study.

Antibody	Host Species	Company	Catalogue Number
ZO-1	Rabbit	Proteintech Group, Inc. (Rosemont, IL, USA)	21773-1-AP
Occludin	Rabbit	Proteintech Group, Inc. (Rosemont, IL, USA)	27260-1-AP
Claudin-5	Rabbit	Affinity Biosciences LTD (Cincinnati, OH, USA)	AF5216
Claudin-11	Rabbit	Abcam Plc (Cambridge, UK)	ab53041
NeuN	Rabbit	Proteintech Group, Inc. (Rosemont, IL, USA)	26975-1-AP
GAD2	Rabbit	Proteintech Group, Inc. (Rosemont, IL, USA)	20746-1-AP
CREB	Rabbit	Abcam Plc (Cambridge, UK)	ab32515
p-CREB	Rabbit	Abcam Plc (Cambridge, UK)	ab32096
BDNF	Rabbit	Proteintech Group, Inc. (Rosemont, IL, USA)	28205-1-AP
ADORA2A	Rabbit	Abcam Plc (Cambridge, UK)	ab3461
GAPDH	Mouse	Proteintech Group, Inc. (Rosemont, IL, USA)	60004-1-Ig

**Table A2:** The oligonucleotide primers used in this study.

mRNAs	Forward 5′→3′ Primer	Reverse 5′→3′ Primer
*β-actin*	GACGGCCAGGTCATCACTATTG	AGGAAGGCTGGAAAAGAGCC
*adcy5*	AGGAAAGTGCGAACCCAGAG	AAGGTCAGGAGGAACTTGCG
*adora2a*	GATGGAGAGCCAACCCCTAC	TTGATGATGTGCAGGGGCAA
*atp2al*	CACCAGCTAAGTCCTCACGG	CAGAACCATCTCCTCGCGTT
*cftr*	TTCTTCACGCCCCTATGTCG	GGGTTGTAATGCCGAGACGA
*cngb1*	CCGGGGTCTTTGCTTTCTCT	TTGCGAATGCCAGGTGTACT
*drd1*	AGGTTGAGCAGGACATACGC	TTGCTTCTGGGCAATCCTGT
*drd2*	GTTTCTGTACCCTTCGGGGG	AGAAGTGGCGGAGTGGATTG
*hcn4*	TGAGCCACTTCGAGAGGAGA	TGGAAGACCTCGAAACGCAA
*htr1b*	ACAGACACCCAACAAGACCG	CCTAGCGGCCATGAGTTTCT
*htr1d*	CGAGCCGTGTGTGTAGAAGT	AAGCCCCTGTCACATTCAGG
*oxt*	CCTGCCCCAGTCTCGCTT	TCCAGGTCCAGCACAGCC
*pde10a*	TGGTGAACAAGATCAGCGGT	GATGCGTGCTGGTAGGTTGA
*ppp1r1b*	AAGTGGAAGGCAGAGCAACA	CAGCCCTAGCAGGTGAAAGA
*sstr5*	CAGGTAGCAGGCAGGAACAA	TTCTGGGTCTGGCGTAGAGA
